# Optimized sensor-embedded loose garment for accurate motion detection

**DOI:** 10.1038/s44172-026-00675-8

**Published:** 2026-05-06

**Authors:** Mohamed Elgendi, Tomasz Raczyński, Alexander Shokurov, Daniel Janczak, Małgorzata Jakubowska, Carlo Menon

**Affiliations:** 1 Department of Biomedical Engineering and Biotechnology, Khalifa University of Science and Technology, Abu Dhabi, UAE; 2 Healthcare Engineering Innovation Group (HEIG), Khalifa University of Science and Technology, Abu Dhabi, UAE; 3 Biomedical and Mobile Health Technology Lab, Department of Health Sciences and Technology, ETH Zürich, Zürich, Switzerland; 4 Institute of Mechanics and Printing, Faculty of Mechanical and Industrial Engineering, Warsaw University of Technology, Warsaw, Poland; 5 Central Laboratory, Centre for Advanced Materials and Technologies (CEZAMAT), Warsaw, Poland

**Keywords:** Biomedical engineering, Electronic properties and materials

## Abstract

Wearable technology has become increasingly important for health monitoring, sports performance, and ergonomic assessments because it enables continuous, non-invasive, and real-time tracking of physiological and biomechanical signals in real-world environments, overcoming limitations of laboratory-based assessments. This paper presents the development, testing, and initial study of a sensor-embedded loose garment designed for motion analysis using conductive ink. Sensors were strategically placed across key areas of the T-shirt to capture comprehensive motion data from the torso. Positioned on the chest, shoulders, ribcage, and lower torso, these sensors detect detailed movements. The study evaluates various sensor combinations with four classifiers—XGBoost, RandomForest, SVM, and K-Nearest Neighbors—using data from ten sensor locations analyzed with three holdout methods (20–80%, 30–70%, and 50–50%). Results underscore the impact of specific sensor placements, with combinations on the shoulder, ribcage, and abdomen yielding the highest accuracy. This work advances textile-based motion recognition, showing the potential for wearable technology to distinguish among eight movements in a loose garment.

## Abstract (web summary)

Smart garments for motion capture are usually tight-fitting, have rigid components, and are expensive. Mohamed Elgendi and colleagues demonstrate a loose t-shirt instrumented with simple printed sensors, whose output is analyzed using machine learning to detect wearer poses and movement.

## Introduction

Wearable technology has emerged as a significant innovation in the fields of health monitoring, sports, and ergonomics, offering a wide array of applications that enhance human performance and well-being^1–3^. In recent years, wearable sensors embedded in textiles have gained considerable attention due to their ability to provide continuous and non-invasive monitoring of physiological and biomechanical parameters^4–6^. The integration of sensors into everyday clothing, such as T-shirts, offers a convenient and unobtrusive method for tracking body movements and vital signs^7–9^.

Recent work in textile-based sensing has highlighted the importance of balancing sensing performance with mechanical compliance and seamless integration into garments. In particular, capacitive textile sensors have demonstrated the need to carefully trade off sensitivity, robustness, and wearability to achieve reliable real-world performance^10^. In parallel, advanced materials such as electrospun nanofibers have been explored to enhance sensitivity and conformability in wearable health monitoring systems^11^. While these approaches offer high-performance sensing capabilities, they often rely on complex fabrication processes or multilayer architectures that may limit scalability and practical deployment.

Recent advancements have also focused on improving the accuracy, miniaturization, and energy efficiency of wearable sensors^12–14^. For instance, wearable inertial sensors have been pivotal in motion analysis, enabling precise gait analysis and movement tracking outside laboratory settings^15,16^. Additionally, ultrasonic-system-on-patch technology has emerged, offering real-time monitoring with enhanced image clarity and reduced false alarms through advanced AI algorithms^17^. These systems are crucial for applications requiring continuous monitoring and real-time data transmission, such as chronic condition management and personalized healthcare^18^.

The development of sensor-embedded garments involves multiple stages, including the selection of suitable sensors, their optimal placement on the garment, and the creation of robust data acquisition systems^19,20^. Conductive inks, such as those made from silver nanoflakes or carbon black, are often used to create flexible and durable sensors that can be seamlessly integrated into fabrics^21,22^. These conductive inks allow for the printing of sensors directly onto textiles, providing a high degree of flexibility and comfort for the wearer^23^.

One of the critical challenges in designing sensor-embedded garments is determining the optimal placement of sensors to capture accurate and reliable data across various activities^24^. Previous studies have shown that the positioning of sensors significantly affects the quality of data collected, which in turn impacts the performance of motion detection and classification algorithms^25,26^. By strategically placing sensors in areas that experience significant distortion during wear, it is possible to enhance the capture of body movements and improve the overall accuracy of the system^27^.

Wearable sensors have been extensively studied for their potential in health monitoring and motion analysis^28^. Previous research has demonstrated the feasibility of using various types of sensors, such as accelerometers, gyroscopes, and electromyography sensors, for capturing different aspects of body movements^29–31^. The integration of these sensors into garments, such as T-shirts, has been explored to provide continuous and unobtrusive monitoring solutions^23^.

Conductive inks and flexible sensors have also been a topic of interest in wearable technology research^32^. Boumegnane et al.^33^ developed a conductive ink using silver nanoflakes for printing sensors on textiles. Their study demonstrated the potential of conductive inks for creating flexible and durable sensors that can withstand repeated washing and stretching. Yang et al.^34^ reviewed various types of flexible sensors and their applications in wearable electronics, highlighting the importance of material properties and sensor design in achieving reliable performance.

Current research in integrated flexible electronics in most cases uses tight-fitting garments with stretchable piezo-resistive sensors, typically positioned on body joints^35^. While this approach allows for accurate movement measurement, it often requires precise positioning of sensors and case by case calibration^36^. By developing a system that can determine posture using sensors embedded in loose garments, we can greatly expand the applicability of this technology, particularly for post-operative patients and the elderly, who might be unable to wear tight-fitting clothes.

In designing and developing textile-based systems, cost-effectiveness and scalability are crucial considerations. It’s important to not only create washable and reusable systems but also lower the manufacturing costs to increase accessibility. For this reason, screen-printing was chosen as the primary manufacturing technique. Screen-printing is a well-known, easily scalable technique that produces flexible sensors with minimal material use, keeping the final sensor cost low^37^. The printed sensors can bend, stretch, and conform to the shape of the fabric, maintaining their functionality even under significant deformation^38^. This flexibility is essential for creating comfortable, unobtrusive devices that can be worn for extended periods of time.

This study builds on the existing body of research by integrating multiple sensors into a single garment and evaluating their performance in motion detection tasks. By systematically analyzing different sensor combinations and classifier performances, we aim to identify the most effective configurations for accurate motion analysis. The findings of this study contribute to the growing field of wearable technology and provide valuable insights for future developments in sensor-embedded garments.

## Methodology

The methodology section outlines the steps taken in the development and testing of the sensor-embedded T-shirt.

### Ethics approval and consent to participate

All experimental procedures involving human participants were reviewed and approved by the ETH Zurich Ethics Commission (IRB00007709). Ethical approval was granted under protocol number EK-2023-E-2 (approved on 04 September 2023). Written informed consent was obtained from all participants prior to their inclusion in the study. Participation was voluntary, and participants were free to withdraw at any time without penalty. The study was conducted in accordance with the principles of the Declaration of Helsinki and complied with all applicable local ethical regulations and institutional review requirements at ETH Zurich.

### Materials

For the manufacturing of the sensors, three different composites were used. The first was an isolating and stabilizing layer made from black plastisol ink TEXPRINT-AQ from Siebdruck Versand, Germany. The sensing layer was composed of a composite containing VXC72R grade carbon black (CB) (Cabot corporation), Poly(methyl methacrylate) (PMMA) and 2-(2-Butoxyethoxy)ethyl acetate (BEA) from Merck, Switzerland. For the connecting pads, a composite was used that included silver flakes of high aspect ratio (Ag) with 10 μm size (Sigma-Aldrich), thermoplastic polyurethane (TPU) Elastollan 1170A from BASF, Germany, N,N-Dimethylformamide (DMF) from Merck, Switzerland and BEA. All sensors were screen-printed on a 100% cotton, size-L t-shirt with 145g/cm2 fabric weight from Creativ Company Ltd (UK). The material and size of the shirt were chosen to be a loose fit to accommodate all the participants in the study. For connecting the sensors to the data acquisition hardware, an insulated conductive thread (Bekaert Bekintex VN 12.3.2.175S.HT.T01.K00E) was used.

For connecting the sensors to the data acquisition hardware, an insulated conductive thread (Bekaert Bekintex VN 12.3.2.175S.HT.T01.K00E) was used. This lightweight, flexible and electrically insulated thread was chosen over conventional wires to minimize tugging or mechanical artifacts during garment movement.

### Protocol for manufacturing of the conductive flexible ink

Both manufactured composites consisted of two main components: a polymer-based vehicle and conductive particles. The vehicle for the sensing layer was created by dissolving 8 %wt of PMMA in BEA for 48 h using magnetic stirrer at the temperature of 40 ^∘^C. To prepare the final composite, a 10 %wt of CB was mixed with the vehicle with a mortar and pestle for 20 min to achieve a homogenous composition. Due to the hygroscopic properties of TPU, before further processing, the TPU pellets were pre-dried at 80 ^∘^C overnight. The vehicle for the contact pads was created by dissolving TPU in a mixture of DMF and BEA, at a weight ratio of 1:3:6 (TPU:DMF:BEA) for 24 h using magnetic stirrer at the temperature of 40 ^∘^C. Due to the fine size of carbon black particles and possible carcinogenic properties (classified by IARC as group 2B), the entire process of composite ink production was performed in a fume hood. After encapsulation of carbon black within the polymer matrix, the resulting composite is generally considered safe for use, provided that the matrix polymer is biocompatible^39–41^. Importantly, all sensors studied in this work were fabricated on the outside of the T-shirt, thereby further minimizing any potential contact with the skin.

### Sensor manufacturing

The textile strain sensors were fabricated by sequential screen printing directly onto a loose-fitting cotton T-shirt using 72T mesh screens and a manual screen-printing setup. The fabrication process involved three functional printed layers: (i) silver composite ink pads forming the electrical contacts, (ii) a piezoresistive carbon-black-based composite ink defining the active sensing region, and (iii) an optional backing layer of commercially available black plastisol ink applied beneath the sensing layer. Each layer was carefully aligned to ensure consistent geometry and was dried for approximately 10 s using a heat gun after printing to promote adhesion and preserve electrical performance.

Electrical connections were established using an insulated conductive thread. The stripped ends of the thread were coated with conductive silver ink and cured at 120 ^∘^C for 20 min before being soldered directly to the silver contact pads, forming a reliable and flexible electrical interface suitable for textile integration.

**Fig. 1:**
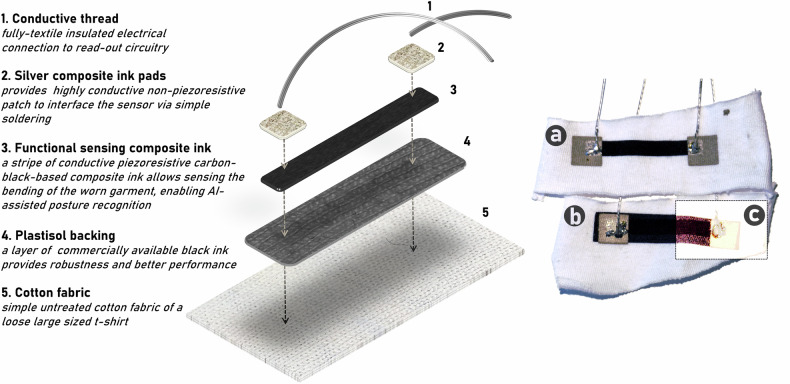
Structure and fabrication of the textile strain sensor. **Left:** Schematic illustration of the layered architecture of the sensor embedded in a loose-fitting cotton T-shirt. The sensor consists of a fully textile insulated conductive thread that provides electrical connection to the data acquisition circuitry, silver composite ink pads forming a highly conductive and non-piezoresistive interface for reliable soldering, and a piezoresistive carbon-black-based composite ink layer that acts as the active sensing element for detecting garment bending. A backing layer of commercially available black plastisol ink is applied beneath the sensing layer to enhance mechanical robustness, improve adhesion to the textile substrate, and stabilize the electromechanical response. The base layer is untreated cotton fabric from a loose, large-sized T-shirt. **Right:** Photographs of standalone textile sensors fabricated according to the process shown in the left panel. Sensor (**a**) is printed without a plastisol backing layer and shows the exposed carbon-black-based sensing ink between two silver contact pads. Sensor (**b**) includes the black plastisol backing, which is visually similar in color to the sensing layer. Inset (**c**) shows a magnified view with enhanced brightness and contrast, allowing clear distinction between the plastisol backing layer and the piezoresistive sensing ink.

A schematic of the layered sensor architecture and representative standalone sensors fabricated with and without the plastisol backing are shown in Fig. 1. In the final sensor design, the piezoresistive sensing layer is positioned between two silver ink pads, forming a rectangular active area of 10 × 60 mm.

**Fig. 2:**
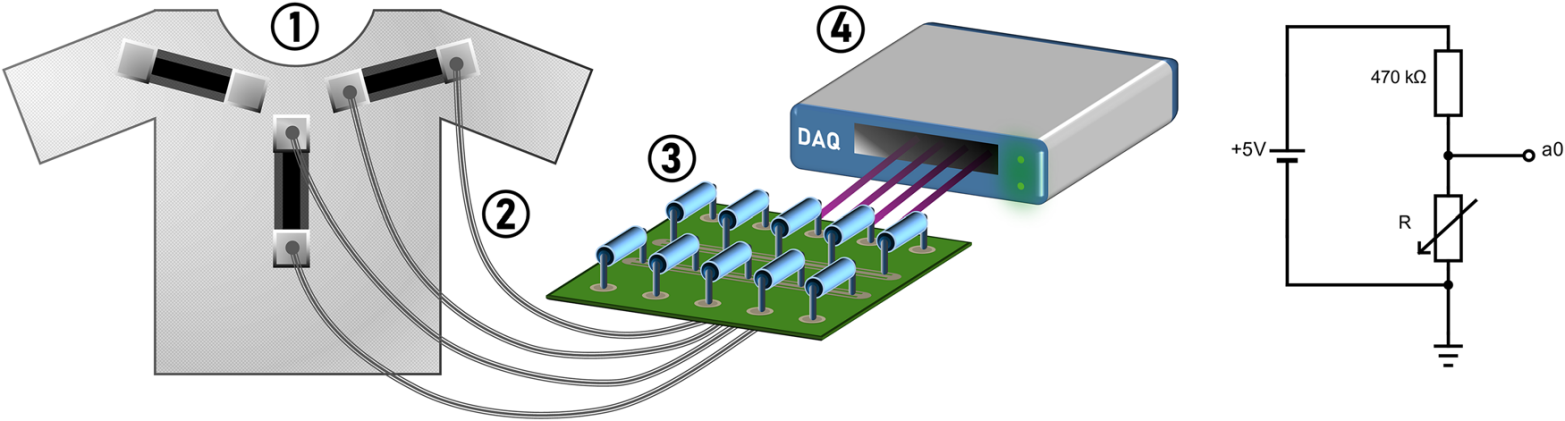
Data acquisition setup for the sensor-embedded loose garment. **Left:** Schematic illustration of the laboratory-based data acquisition system (not to scale). (1) A loose T-shirt with printed resistive sensors worn by the participant, where body movements induce sensor deformation and corresponding resistance changes. (2) Each sensor terminal is connected using lightweight, insulated conductive yarn, providing a flexible alternative to rigid wiring and minimizing mechanical interference during motion. (3) A simple passive voltage divider circuit used to convert sensor resistance changes into measurable voltage signals. (4) A multifunction data acquisition (DAQ) device with analog and digital I/O that applies the interrogating voltage, acquires sensor output voltages, and streams the data to a computer for storage and analysis. **Right:** Schematic of the voltage divider circuit used for resistive readout, where *R* denotes the variable resistance of the printed sensor.

Printed sensors were then connected to the data acquisition setup by carefully soldering the insulated conductive yarn to the silver-based terminals of each sensor. The other ends of the yarns were connected to a read-out circuit board: a simple voltage divider circuit consisting of a 470 k*Ω* through-hole resistor matched to each sensor. The board was connected to a multifunctional data acquisition device (National Instruments USB X Series DAQ, NI USB-6341), which applied a 5 V excitation and sampled each channel at 20 Hz. Data were recorded in LabVIEW as synchronized time series. The overall data acquisition setup and circuit configuration are illustrated in Fig. 2.

The present data acquisition setup is laboratory-based and was not designed to be wearable or wireless in its current form. No post hoc correction or compensation for potential wire-induced artifacts was applied; instead, the machine-learning models were trained directly on the acquired signals, implicitly accounting for any residual effects present in the data.

### Sensor placement

**Fig. 3:**
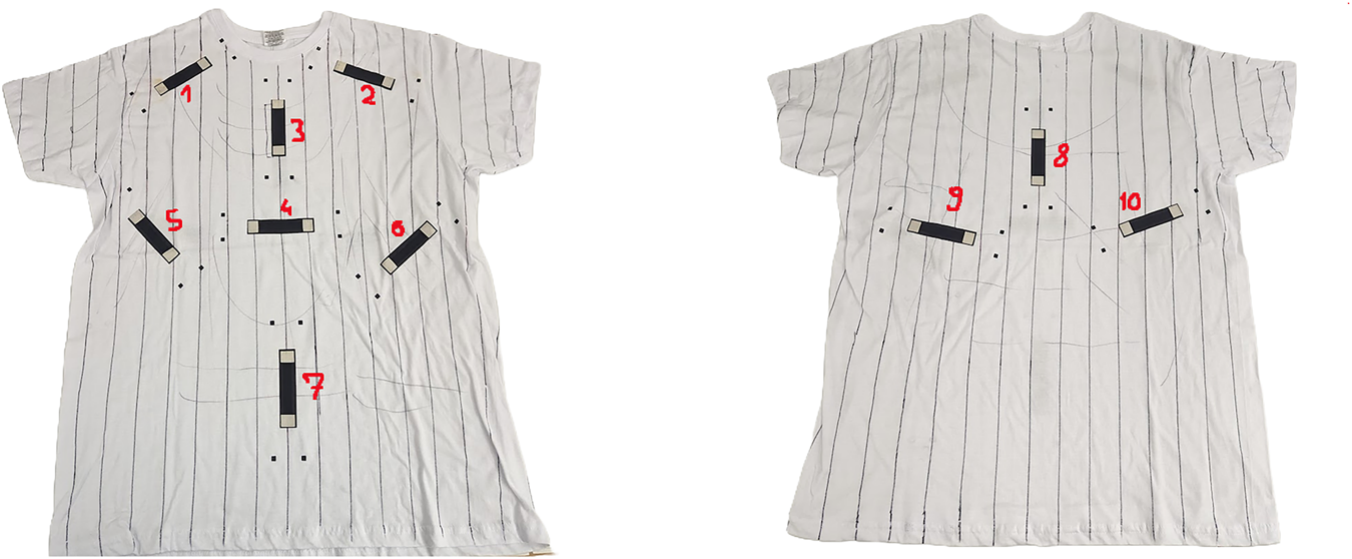
Sensor placement. This figure shows the sensor placement on the T-shirt, with sensors labeled from 1 to 10. The front view (left) and back view (right) display the locations selected based on areas of the T-shirt that experience the most distortion during wear. These placements were determined to optimize the capture of body movements and ensure reliable data collection.

The sensor placement on the T-shirt is illustrated in Fig. 3, which shows both the front and back views of the T-shirt. Sensors were strategically placed in areas that experience significant distortion during wear to optimize the capture of body movements. The front view includes sensors labeled 1 through 7, while the back view includes sensors labeled 8 through 10. This placement was determined based on preliminary tests and analysis, ensuring that the sensors would provide accurate and reliable data across a range of activities. By positioning the sensors in these key areas, the T-shirt can effectively monitor and analyze body movements, making it a valuable tool for motion analysis and wearable technology applications.

On the front of the T-shirt, Sensor 1 is located on the right shoulder area, positioned horizontally to capture shoulder movements. Similarly, Sensor 2 is positioned on the left shoulder area, also horizontally aligned. These shoulder sensors are crucial for monitoring upper body movements and detecting shoulder displacements. Sensor 3 is centrally positioned along the chest, placed vertically to monitor chest expansion and contraction during breathing and other activities. Sensor 4 is located horizontally in the middle of the torso, near the diaphragm, to capture breathing movements and diaphragm activity. Additionally, Sensors 5 and 6 are placed diagonally on the right and left sides of the torso, respectively, near the ribcage. These sensors are intended to capture lateral torso movements and provide data on side-to-side displacements. Sensor 7 is positioned vertically in the lower center of the torso, near the abdomen, to capture movements involving the lower torso and abdominal region, which is particularly useful for analyzing movements such as bending and twisting.

On the back of the T-shirt, Sensor 8 is centrally positioned along the upper back, vertically aligned to capture upper back movements. This sensor is critical for monitoring the spine and upper back activities. Sensors 9 and 10 are placed diagonally on the right and left sides of the upper back, respectively, near the shoulder blades. These sensors are designed to capture scapular movements and provide detailed data on shoulder blade activity. This strategic placement of sensors ensures comprehensive motion analysis by capturing data from various key points on the torso, thereby enhancing the effectiveness of the T-shirt for applications in motion analysis and wearable technology.

### Data collection protocol

A new sensor design was developed to facilitate easier positioning of the different sensor layers. A prototype T-shirt incorporating ten sensors was created. An initial study protocol for the T-shirt using human participants was devised and later adjusted to include additional asymmetric movements. This final study protocol was used to conduct measurements with three participants.

The protocol was structured as follows: each position was maintained for a duration of 20 s. Each transition between positions was repeated five times to ensure consistency and reliability of the data. The entire protocol was divided into seven sets, with each set focusing on transitions from Position 1 to other positions. The specific transitions recorded were: Set 1: Position 1 to Position 2, Set 2: Position 1 to Position 3, Set 3: Position 1 to Position 4, Set 4: Position 1 to Position 5, Set 5: Position 1 to Position 6, Set 6: Position 1 to Position 7, and Set 7: Position 1 to Position 8. In this context, each “set” refers to a group of transitions starting from Position 1 to one of the other positions (Positions 2–8). By dividing the protocol into these sets, we ensure that each specific movement transition is captured thoroughly and consistently across multiple repetitions.

**Fig. 4:**
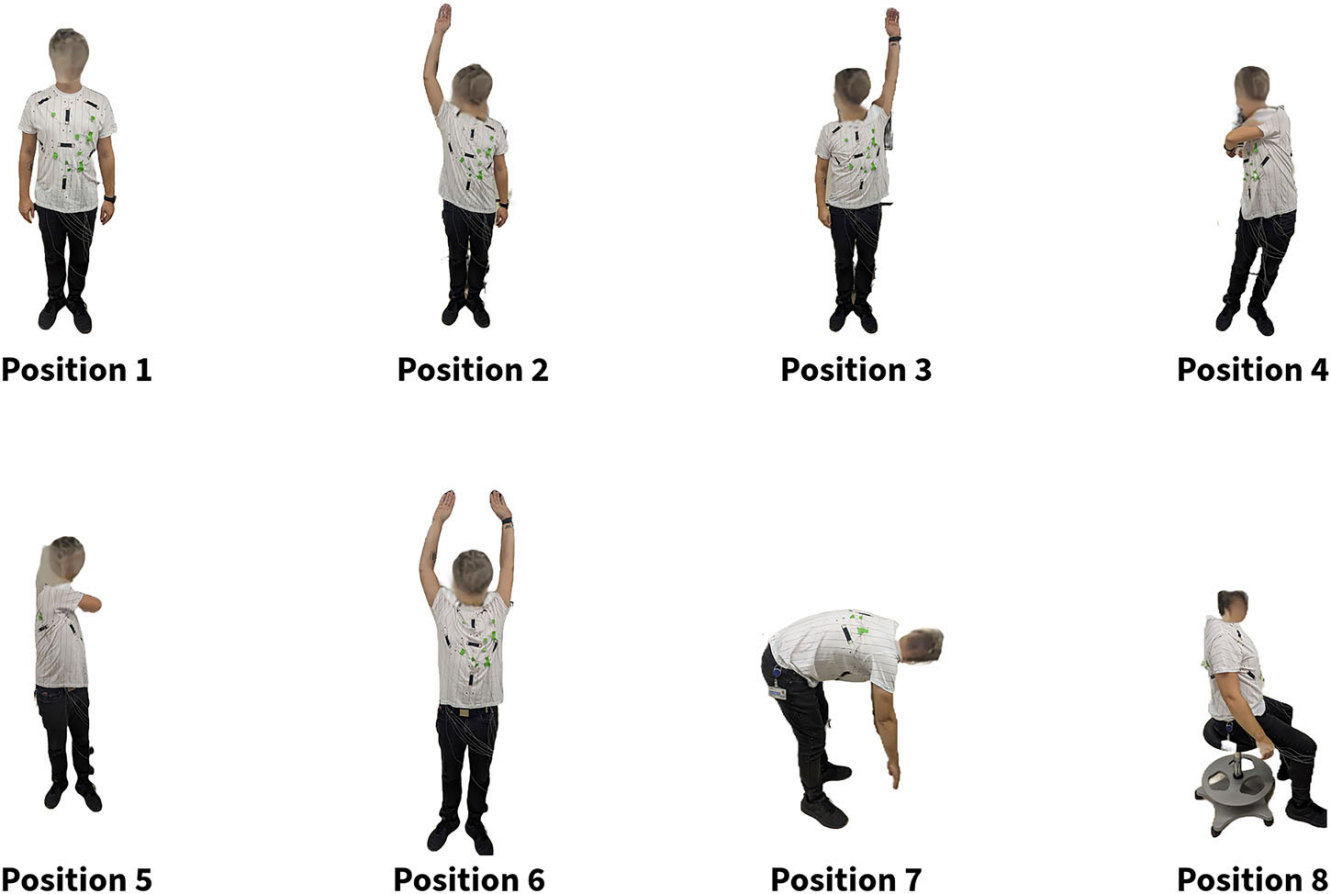
Positions captured by the sensor-embedded T-shirt. This figure shows eight different positions used during the study to determine the optimal areas for sensor placement on the T-shirt. The positions are as follows: (1) standing straight, (2) raising the left arm, (3) raising the right arm, (4) crossing the arms, (5) touching the left shoulder, (6) raising both arms, (7) bending forward, and (8) sitting. These positions help identify areas of the T-shirt that experience the most distortion during wear.

Figure 4 illustrates the sensor data collected over time for each position, providing a detailed overview of the variations in sensor readings during the transitions.

These positions were carefully selected to represent a range of common and asymmetric movements that would challenge the sensor system’s ability to accurately capture and distinguish different motions. The positions include standing straight (Position 1), raising the left arm (Position 2), raising the right arm (Position 3), crossing the arms (Position 4), touching the left shoulder (Position 5), raising both arms (Position 6), bending forward (Position 7), and sitting (Position 8). By incorporating these diverse movements, the study aimed to identify areas of the T-shirt that experienced the most distortion, thereby optimizing sensor placement and ensuring reliable data collection across various activities.

A software in LabView was developed, and hardware circuitry was soldered to allow data collection from the instrumented T-shirt using DAQ. Successful measurements with a single sensor led to scaling the system to collect data from all ten sensors.

All experiments were conducted indoors under controlled laboratory conditions. The effects of environmental factors such as humidity and temperature on sensor stability were not explicitly evaluated in this study. Investigating sensor performance under varying humidity, temperature, and repeated washing conditions will be the focus of future work.

### Participants of the study

All the data for the T-shirt with printed sensors was collected with three participants: two males and one female. All participants reported normally wearing size M. They were provided with an XL size T-shirt with printed sensors to guarantee a loose fit. Although anthropometric parameters such as height, weight, or BMI were not recorded, the garment choice was intentionally oversized to ensure that the sensors were tested under “loose garment” conditions. Future studies will incorporate a larger cohort and include these measurements for more detailed analysis.

### Data processing and analysis

The data collected from the sensors, represented as raw signals *x*_*i*_(*t*) for each sensor *i*, underwent *z*-score normalization to ensure consistency and comparability across different sensor readings. Importantly, the normalization parameters (mean *μ*_*i*_ and standard deviation *σ*_*i*_) were computed on the *training split only* to avoid data leakage, and the same parameters were then applied to normalize the corresponding test split:

1 $${x}_{n,i}(t)=\frac{{x}_{i}(t)-{\mu }_{i}}{{\sigma }_{i}}.$$

This ensures standardization of the sensor data while preserving the integrity of the train-test evaluation.

### Statistical analysis

Statistical analysis was performed to evaluate the ability of the sensor-embedded T-shirt to discriminate between eight different body positions. One-way Analysis of Variance (ANOVA) was employed to test whether statistically significant differences existed in the sensor signals across positions, following standard statistical practice^42^. ANOVA compares the means of multiple groups and tests the null hypothesis that all group means are equal.

The F-statistic used in the ANOVA is defined as

2 $$F=\frac{\,{{{\rm{Between}}}} {\mbox{-}} {{{\rm{position}}}}\; {{{\rm{variability}}}}}{{{{\rm{Within}}}} {\mbox{-}} {{{\rm{position}}}}\; {{{\rm{variability}}}}}=\frac{\frac{{\sum }_{i=1}^{k}{n}_{i}{({\bar{x}}_{i}-\bar{x})}^{2}}{k-1}}{\frac{{\sum }_{i=1}^{k}{\sum }_{j=1}^{{n}_{i}}{({x}_{ij}-{\bar{x}}_{i})}^{2}}{N-k}},$$

where *k* denotes the number of positions, *n*_*i*_ is the number of observations for position *i*, *x*_*ij*_ represents the *j*-th sensor observation belonging to position *i* (*j* = 1, …, *n*_*i*_), ${\bar{x}}_{i}$ is the mean sensor value for position *i*, $\bar{x}$ is the overall mean across all positions, and *N* is the total number of observations. The use of one-way ANOVA for comparing sensor responses across multiple positions follows standard statistical practice in biomedical signal analysis^42^.

A small *p*-value (typically *p* < 0.05) indicates that the observed differences between positions are unlikely to have occurred by chance, suggesting a statistically significant effect of body position on the sensor response.

To further characterize relationships between sensor signals, pairwise Pearson correlation analysis was conducted^43^. The correlation coefficient *r* between two time-indexed sensor signals *x* and *y* is defined as

3 $$r=\frac{\sum ({x}_{i}-\bar{x})( \, {y}_{i}-\bar{y})}{\sqrt{\sum {({x}_{i}-\bar{x})}^{2}\sum {( \, {y}_{i}-\bar{y})}^{2}}}.$$

This analysis provides insight into sensor redundancy and complementarity by quantifying the degree to which sensor signals co-vary across positions, thereby informing sensor selection and layout optimization for position classification.

### Sensor optimization and classification analysis

To evaluate the performance of different sensor combinations, we generated all possible combinations of one, two, and three sensors. We employed four different classifiers: XGBoost, Random Forest, Support Vector Machine (SVM), and K-Nearest Neighbors (KNN). These classifiers were chosen for their ability to handle complex, non-linear relationships in the data and their proven effectiveness in classification tasks^44–47^.

Given the small sample size of three subjects, it was essential to train classifiers separately for each subject. Combining all subjects’ data into a single classifier increases the risk of overfitting, where the model learns noise and specific details rather than underlying patterns, resulting in poor generalization. Training classifiers separately mitigates this risk, as each classifier is exposed to a more homogeneous dataset, making it easier to identify consistent patterns within each subject’s data. This approach ensures robust and reliable identification of the most effective sensor configurations, tailored to each individual, without being influenced by inter-subject variability.

Let *C*_*k*_ represent a combination of *k* sensors, where *k* ∈ {1, 2, 3}. The accuracy *A* for each combination and classifier was calculated using the following methodology:

First, the dataset *D* was split into training *D*_train_ and testing *D*_test_ sets using the holdout methods. For a 20–80% holdout, 20% of the data was used for testing and 80% for training, denoted as:*D* = *D*_train_ ∪ *D*_test_, 7D1∣*D*_test_∣ = 0.2∣*D*∣, 7D1∣*D*_train_∣ = 0.8∣*D*∣. 

Each classifier ${f}_{{C}_{k}}$ was then trained on the training set *D*_train_ with sensor combination *C*_*k*_: ${f}_{{C}_{k}}({D}_{{{{\rm{train}}}}}).$ The XGBoost classifier *f*_XGB_ optimizes a regularized objective function that combines a convex loss function *L* and a regularization term *Ω*:

4 $${f}_{{{{\rm{XGB}}}}}({D}_{{{{\rm{train}}}}})=\arg \min {\sum}_{i=1}^{n}L({y}_{i},{\widehat{y}}_{i})+{\sum}_{k=1}^{K}\Omega ({f}_{k}),$$

where *D*_train_ represents the training dataset, *y*_*i*_ and ${\widehat{y}}_{i}$ are the true and predicted labels respectively, *f*_*k*_ denotes the individual trees, and *K* is the number of trees.

The Random Forest classifier *f*_RF_ constructs an ensemble of decision trees ${\{{T}_{k}\}}_{k=1}^{K}$, where each tree *T*_*k*_ is trained on a bootstrap sample of the training data with feature subsampling. For multi-class classification, the forest prediction is obtained by averaging the trees’ class-posterior estimates and taking the argmax:

5 $$\widehat{p}(c| x)=\frac{1}{K}{\sum}_{k=1}^{K}{\widehat{p}}_{k}(c| x),\,\widehat{y}(x)=\arg {\max }_{c\in {{{\mathcal{C}}}}}\widehat{p}(c| x),$$

where ${\widehat{p}}_{k}(c| x)$ is the class proportion at the leaf reached by *x* in tree *k*, and ${{{\mathcal{C}}}}$ is the set of classes. The Support Vector Machine (SVM) classifier *f*_SV M_ finds the maximum-margin hyperplane *w*^⊤^*x* + *b* = 0 in the soft-margin setting by solving

6 $${\min }_{w,b}\,\frac{1}{2}\parallel w{\parallel }^{2}\,+\,C{\sum}_{i=1}^{n}\max \left(0,\,1-{y}_{i}\left({w}^{\top }{x}_{i}+b\right)\right),$$

where ${x}_{i}\in {{\mathbb{R}}}^{d}$ are training vectors, *y*_*i*_ ∈ { − 1, + 1} the class labels, and *C* > 0 the regularization parameter. In practice, we use a kernelized SVM and one-vs-rest for multi-class classification.

The *k*-Nearest Neighbors (KNN) classifier predicts by majority vote among the *k* closest training samples ${{{{\mathcal{N}}}}}_{k}(x)$ under a chosen distance (e.g., Euclidean):

7 $$\widehat{y}(x)\,=\,\arg {\max }_{c\in {{{\mathcal{C}}}}}{\sum }_{j\in {{{{\mathcal{N}}}}}_{k}(x)}{{{\bf{1}}}}\left({y}_{j}=c\right).$$

where *x* is the input feature vector, ${{{\mathcal{C}}}}$ is the set of possible class labels, *y*_*j*_ are the labels of the *k* nearest neighbors, and ${\mathbb{1}}$ is the indicator function.

Data was collected from ten sensors placed at strategic locations on the T-shirt as described earlier, and various holdout methods (20–80%, 30–70%, and 50–50%) were used to evaluate the performance of each sensor combination^48^. Each classifier ${f}_{{C}_{k}}$ was trained and evaluated on each sensor combination *C*_*k*_, and the accuracy ${A}_{{C}_{k}}$ was recorded. The primary metric for evaluating the performance of the classifiers was accuracy. The average accuracy ${\bar{A}}_{{C}_{k},h}$ for each sensor combination *C*_*k*_ was calculated across all subjects to identify the top sensor combinations.

The trained classifier was subsequently used to predict the labels for the testing set *D*_test_. For a multi-class classification problem with *M* positions, the accuracy ${A}_{{C}_{k}}$ is calculated as the ratio of correctly predicted instances *n*_correct_ to the total number of instances *n*_total_:

$${A}_{{C}_{k}}=\frac{1}{{n}_{{{{\rm{total}}}}}}{\sum}_{i=1}^{{n}_{{{{\rm{total}}}}}}\delta ({\widehat{y}}_{i},{y}_{i}),$$

where ${\widehat{y}}_{i}$ is the predicted label, *y*_*i*_ is the true label, and $\delta ({\widehat{y}}_{i},{y}_{i})$ is the Kronecker delta function that returns 1 if the arguments are equal and 0 otherwise.

The average accuracy ${\bar{A}}_{{C}_{k},h}$ for each sensor combination and holdout method *h* was computed to identify the top-performing combinations:

$${\bar{A}}_{{C}_{k},h}=\frac{1}{N}{\sum}_{i=1}^{N}{A}_{{C}_{k},h}^{(i)},$$

where *N* is the number of subjects, and *h* represents the holdout method (20–80%, 30–70%, or 50–50%). The overall average accuracy ${\bar{A}}_{{C}_{k}}$ across all holdout methods was calculated to provide a comprehensive evaluation of each sensor combination:

$${\bar{A}}_{{C}_{k}}=\frac{1}{H}{\sum}_{h=1}^{H}{\bar{A}}_{{C}_{k},h},$$

where *H* is the total number of holdout methods. By systematically evaluating each sensor combination and classifier, we aimed to identify the most effective configurations for accurate motion analysis.

The overarching goal of this study was to minimize the number of required sensors while maintaining high classification accuracy. We therefore evaluated single sensors, pairs, and triplets systematically. Classification accuracy increased markedly from single sensors to pairs and then to triplets, where it stabilized at consistently high values (above 90% in most cases). Preliminary checks with four or more sensors did not show meaningful improvements over the triplet level, while adding additional sensors would increase garment complexity, manufacturing cost, and reduce user comfort. For these reasons, we limited our detailed analysis to up to three sensors, representing a practical balance between accuracy and minimal instrumentation.

Here, ${n}_{{{{\rm{correct}}}}}={\sum }_{i=1}^{{n}_{{{{\rm{total}}}}}}\delta ({\widehat{y}}_{i},{y}_{i})$ denotes the total number of correctly classified time samples, where the predicted label matches the ground-truth position label, and *n*_total_ corresponds to the number of samples in the evaluated test split.

## Results

### Sensor development

The primary objective of this study was to test the hypothesis that a modern machine-learning approach could be used to accurately recognize a user’s motions using an everyday, non-stretchable, loose-fitting garment instrumented with simple and inexpensive fully-textile sensors. A plain white cotton t-shirt of a large size as a base garment was chosen for that reason. However, while the garment choice was straightforward the choice of the appropriate sensor required more consideration.

For the purpose of the study, it was essential to develop a sensor that can be integrated with clothing, offering flexibility and stretchability (to the extent that relatively inelastic cotton is), resistance to mechanical wear over time and ease of production while functioning effectively as a deformation sensor. For these reasons, a screen-printed resistive sensor was chosen. Screen printing allows for scalability of production, ease of application to existing garments, and compatibility with nearly all textiles. The materials that were chosen for fabrication of the sensors are also readily available, do not modify the fabric they are placed on from the mechanical behavior standpoint, and provide excellent functional properties. The sensors were produced on the t-shirt, as described in the methods section. To evaluate the viability of the design, the sensors were tested by measuring resistance during cyclic bending over the period of 1000 cycles to a bending radius of approximately 5 mm. Each sensor type was tested under two conditions: bending it inwards and outwards (meaning the bending force applied to the printed side of the textile, or from the pristine reverse side, respectively) to test different behaviors of the final setup.

**Fig. 5:**
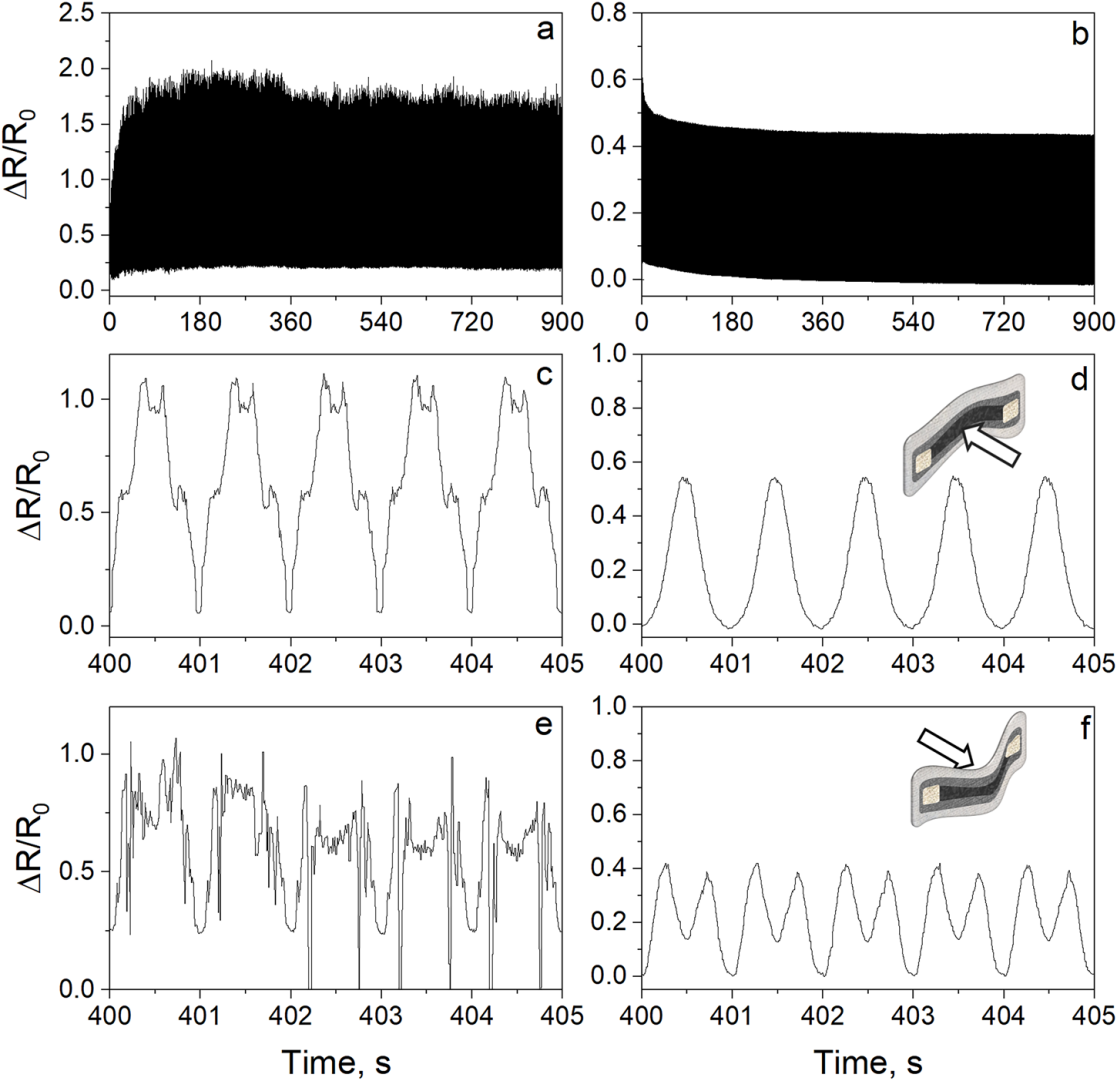
Representative normalized resistance changes (Δ*R*/*R*_0_) of different sensors during cyclic bending. **a**,**c**,e Sensors without plastisol backing. **b**, **d**, **f** Sensors with plastisol backing. Unbacked sensors exhibit systematic artifacts and low reproducibility across cycles, whereas backed sensors show more stable and repeatable waveforms. Both cases exhibit a small transient drift at the start of cyclic testing, which stabilizes after a few cycles.

The first set of samples was produced by directly screen-printing the sensing layer onto the textile. The resulting output, shown in Fig. 5a, exhibited large irregularities in amplitude, rendering the sensor nearly unusable in practical applications where additional noise sources would only exacerbate the problem.

To improve performance, a commercially available black plastisol ink—typically used for artistic screen prints—was added as a backing layer beneath the sensor. This layer enhanced the textile’s mechanical strength and optimized the surface for better adhesion of subsequent layers. Tests following this modification demonstrated substantial improvements, as shown in Fig. 5b. In both configurations, a slight transient drift was observed at the beginning of cyclic bending, but it stabilized after a few minutes. Importantly, unbacked sensors (Fig. 5a) produced irregular waveforms with inconsistent amplitudes, especially at full bending, whereas backed sensors (Fig. 5b) yielded reproducible and stable peaks across cycles, resulting in more reliable data for subsequent analysis (Fig. 5c–f).

To improve signal quality, a commercially available black plastisol ink—typically used for artistic screen prints—was added as a backing layer beneath the sensor. This backing layer enhanced the sensor’s mechanical strength and optimized the t-shirt surface for better adhesion of subsequent layers^49^. Tests following this modification demonstrated significant improvements in signal quality, as shown in Fig. 5b. The backing layer also reduced measurement noise, resulting in more reliable data (Fig. 5c, d).

A significant finding in our study was the contrast in performance between sensors with and without backing. While unbacked sensors were easier and faster to manufacture, their resistance-change signals during cyclic bending contained considerable systematic errors. The shape of the signal varied widely across sensors and cycles, limiting reproducibility (Fig. 5c,e). In contrast, backed sensors produced cleaner and more repeatable waveforms, especially during inward bending (Fig. 5d). The addition of the backing layer reduced variability and ensured more predictable signal shapes, thereby making the collected data more suitable for robust classification.

Moreover, the sensors demonstrated distinct behaviors when bent inwards versus outwards. In particular, outward bending produced a characteristic valley in the middle of the signal peak (Fig. 5e, f). Such waveform differences indicate that the algorithm can distinguish between types of deformation based on signal characteristics alone. However, valleys observed during inward bending may sometimes be misinterpreted as double-frequency signals. To mitigate this risk, classification relies on data from multiple sensors placed at different positions. This redundancy allows the algorithm to use signals with clearer peaks as a reference “clock,” thereby improving robustness. While the double-frequency issue is noteworthy, it is unlikely to affect posture classification in practice, since postures involve relatively static positions rather than rapid oscillations.

The final sensor design consisted of three layers: a backing layer, a sensing layer, and connecting pads, as shown in Fig. 1. This structure ensures that sensors behave in a predictable and consistent way, providing the required sensitivity to deformation while also showing lower variability in signal amplitude.

All cyclic bending responses in Fig. 5 are reported as normalized resistance change (Δ*R*/*R*_0_) rather than absolute resistance values. This normalization removes sensor-to-sensor baseline differences and enables direct comparison of signal amplitudes and waveform shapes across sensors and subjects. The observed differences in waveform shape therefore reflect true differences in local garment deformation, sensor orientation, and bending direction, rather than absolute resistance offsets.

**Fig. 6:**
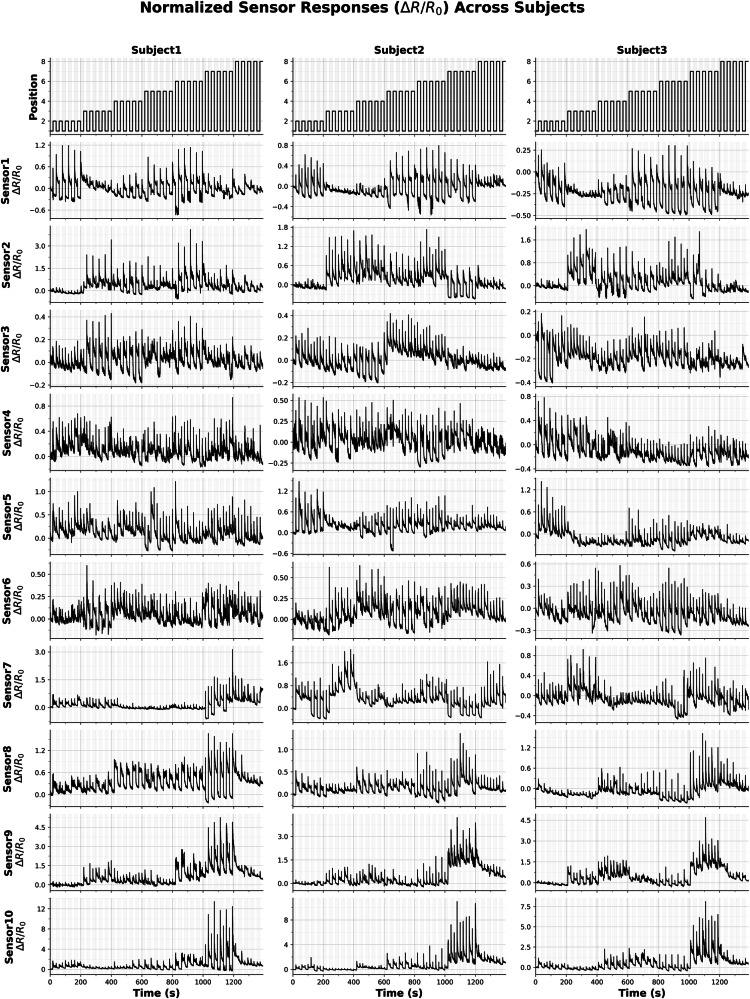
Normalized sensor responses (Δ*R*/*R*_0_) across subjects during repeated positional transitions. The top row shows the discrete position index over time for each subject, indicating successive gesture or posture changes. For each subject, subsequent rows display the normalized resistance change of ten textile sensors, where *R*_0_ is defined as the median resistance during the initial baseline period (first 2 s). Shaded regions indicate alternating gesture segments inferred from position transitions. Across subjects, sensors exhibit distinct temporal response patterns, with certain sensors demonstrating higher sensitivity and dynamic range during specific positional states, highlighting inter-sensor and inter-subject variability.

Consistent with this interpretation, Fig. 6 shows that several sensors (e.g., Sensors 3 and 5–8) exhibit position-dependent variations in waveform shape across subjects despite normalization, confirming that these effects arise from mechanical and geometrical factors rather than from baseline resistance variability.

After development of the sensors that will match the required mechanical, electrical and behavioral properties, successful measurements were obtained with the ten-sensor prototype. Data collected from the T-shirt study involving three participants showed promising results. Asymmetric movements, such as raising a single arm, were easily distinguishable. The time series data *x*(*t*) for all ten sensors and the corresponding positions for each of the three subjects are shown in Fig. 6.

This comprehensive visualization allows for a detailed comparison of sensor responses across different subjects and positions. Each column in the figure represents one subject (Subject 1, Subject 2, and Subject 3), and the rows display the data for each of the ten sensors along with the position data at the top. The position data, indicated in the top row for each subject, shows the changes in position over time, providing context for the sensor readings.

The sensor data *x*(*t*) for each subject shows visually distinct patterns corresponding to different positions. For example, Sensor 1 and Sensor 2, located on the right and left shoulders respectively, exhibit significant changes in resistance values when the arms are raised or lowered. Similarly, Sensor 9 and Sensor 10, located on the upper back, show notable variations during movements involving the upper body. Figure 6 highlights the differences in sensor responses due to individual variations and the specific movements performed by each subject. It underscores the importance of analyzing multiple sensors *C*_*k*_ simultaneously to capture the complexity of human motion accurately. By comparing the sensor data across subjects, we can identify consistent patterns and anomalies, which are crucial for developing robust algorithms for position detection.

**Fig. 7:**
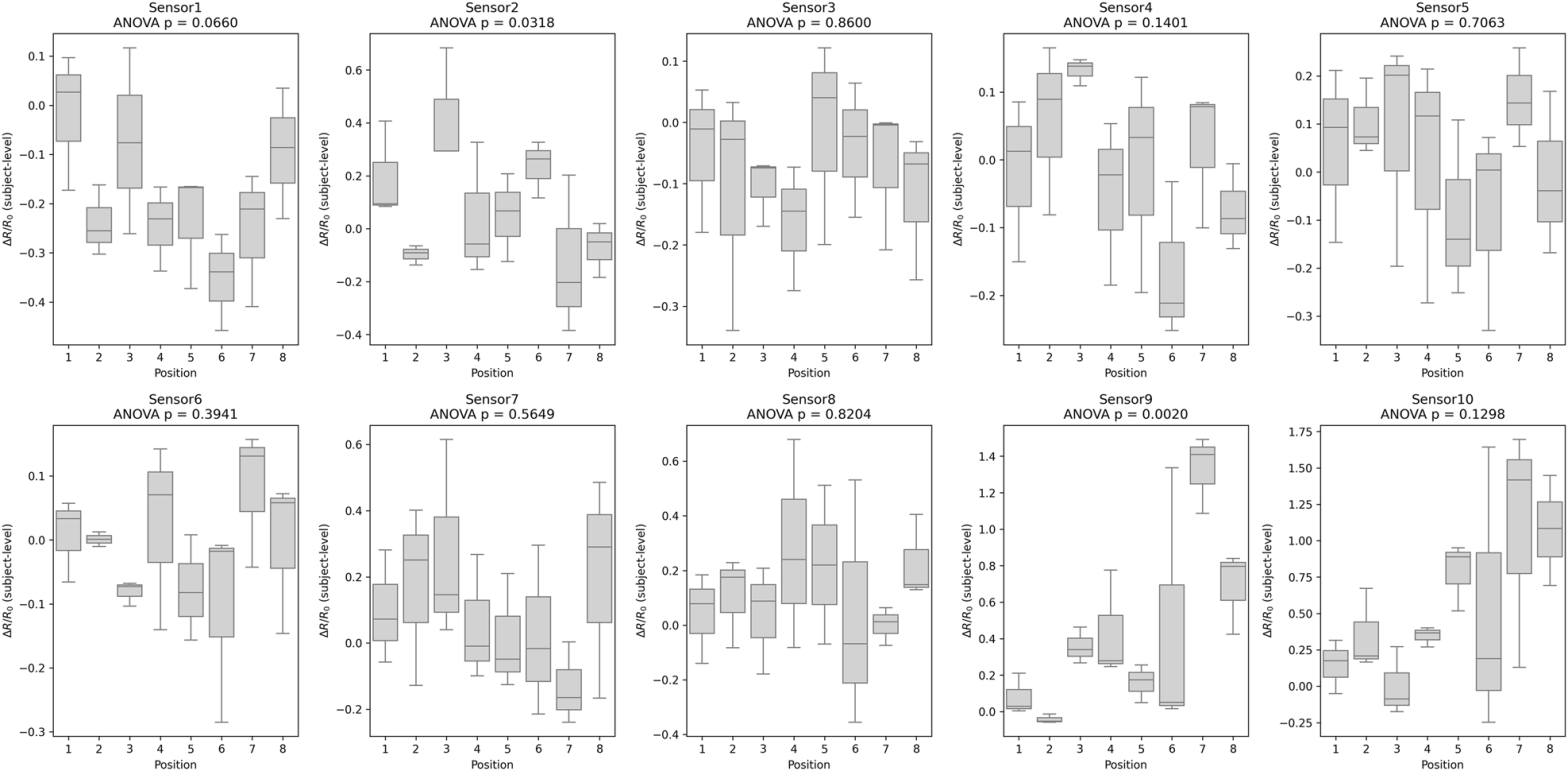
Boxplots of subject-level normalized sensor responses (Δ*R*/*R*_0_) across the eight positions. For each subject and position, the median sensor response over time was computed to ensure statistical independence. Each subplot corresponds to one of the ten sensors. The accompanying one-way ANOVA *p*-values indicate position-dependent differences at the subject level, highlighting Sensors 2 and 9 as exhibiting statistically significant effects, with Sensor 1 showing a borderline trend.

After visually identifying distinct patterns in the sensor data, we quantified position-dependent differences using a one-way ANOVA performed on subject-level summary metrics. To avoid inflated statistical significance due to temporal autocorrelation, the analysis was conducted on per-subject median values of the normalized sensor response (Δ*R*/*R*_0_) for each position. Figure 7 shows the resulting boxplots and corresponding ANOVA *p*-values.

The one-way ANOVA results reveal sensor-specific sensitivity to body position at the subject level. In particular, Sensors 2 and 9 exhibit statistically significant differences across the eight positions (*p* < 0.05), indicating strong and consistent position-dependent responses across participants. Sensor 1 shows a borderline trend toward significance (*p* = 0.066), suggesting moderate sensitivity to postural changes. In contrast, the remaining sensors do not reach statistical significance at the subject level, reflecting either reduced positional sensitivity or increased inter-subject variability.

Although sample-level signal variations are visually evident across most sensors, subject-level statistical testing provides a more conservative assessment of positional sensitivity by explicitly accounting for inter-subject variability. This distinction highlights that not all visually responsive sensors contribute equally to robust, subject-consistent position discrimination.

These results indicate that only a subset of sensors exhibits strong and reliable position-dependent responses at the subject level, while others show weaker or more variable effects across participants. This finding underscores the importance of sensor selection and supports the subsequent machine-learning analysis, where combinations of complementary sensors are leveraged to improve classification performance. Rather than requiring all sensors to be individually significant, the results suggest that effective posture classification can be achieved through the integration of a small number of informative sensors, combined with data-driven feature extraction and classification models.

**Fig. 8:**
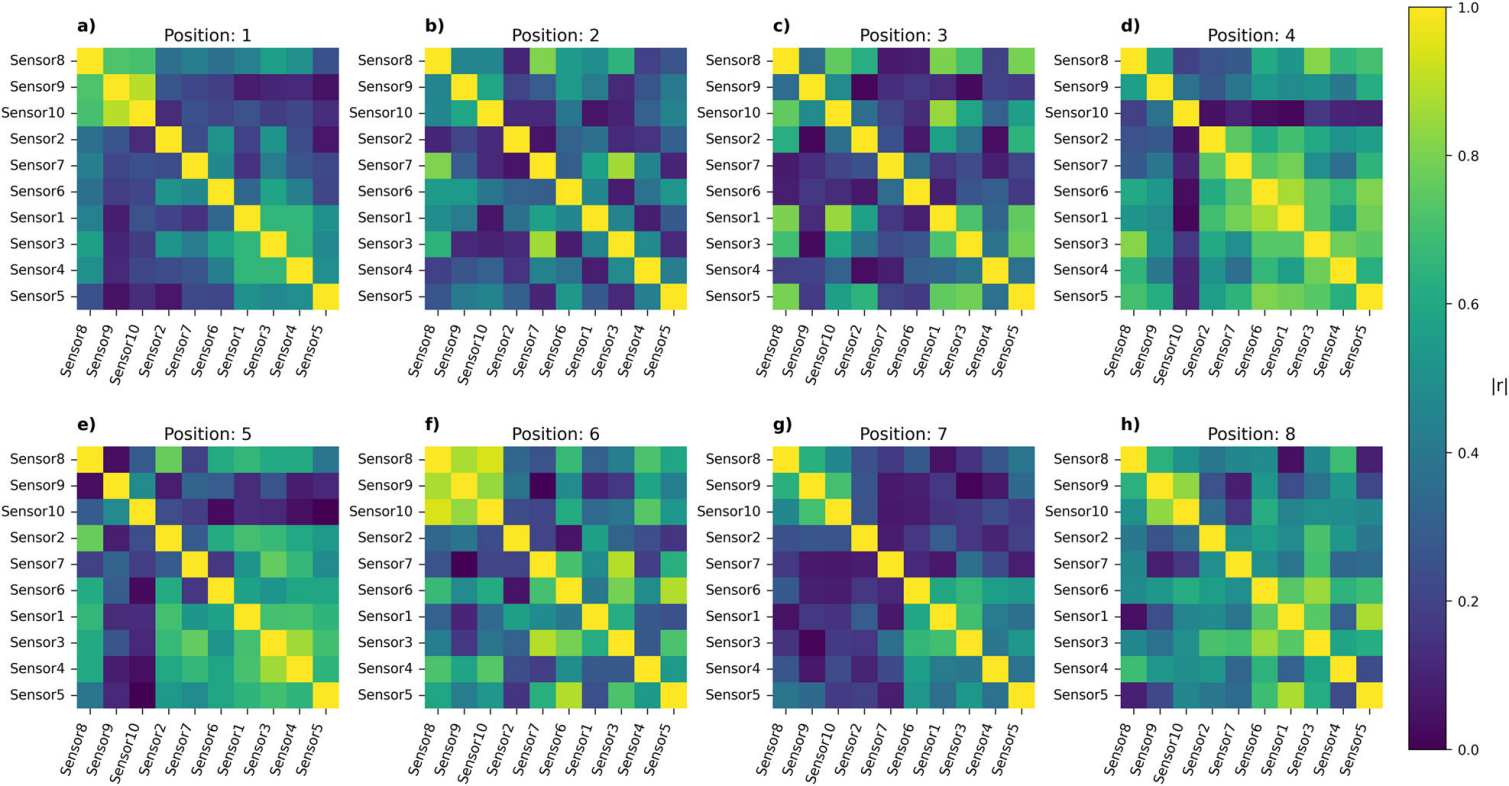
Position-wise sensor correlations. Heatmaps show the absolute Pearson correlation ∣*r*∣ between all sensor pairs computed from normalized signals Δ*R*/*R*_0_ and pooled across subjects. **a-h** correspond to Positions 1–8; a shared color scale spans 0–1. Sensors are ordered consistently across panels (hierarchical clustering on the full dataset) to aid comparison. Higher ∣*r*∣ indicates shared/ redundant signal content, whereas lower ∣*r*∣ suggests complementary information across the array, with position-dependent patterns (stronger bilateral coupling in symmetric postures and lateralization in asymmetric ones).

Figure 8a-h reports position-wise pairwise correlations between the ten sensors, computed on normalized signals (Δ*R*/*R*_0_) and pooled across subjects. For readability, we show the absolute Pearson coefficient, ∣*r*∣ (0–1), and keep a fixed sensor ordering across panels (derived from hierarchical clustering on the full dataset). In this representation, higher ∣*r*∣ denotes shared or potentially redundant information, whereas lower ∣*r*∣ indicates complementary sensing.

Across positions (Fig. 8a-h) three consistent features emerge. *First*, sensors located on nearby or mirrored anatomical regions tend to form coherent correlation “blocks,” visible as brighter off-diagonal patches. These blocks strengthen in symmetric postures and weaken in asymmetric ones, reflecting bilateral loading versus lateralised strain. *Second*, the strength and geometry of these blocks are position-dependent: some postures concentrate coupling within a small subset of sensors, whereas others distribute correlations more broadly. *Third*, several channels retain low to moderate correlations across most panels, suggesting they capture distinctive mechanical information even when other sensors covary.

Sensor-specific patterns further support these observations. Sensor 10 shows strong coupling with Sensors 8 and 9 (∣*r*∣ = 0.78 and 0.67, respectively), consistent with their proximal placement and similar loading; by contrast, its correlations with Sensors 1 and 2 are weak (∣*r*∣ = 0.21 and 0.02), indicating complementary information content. Sensor 1 likewise exhibits only moderate links to the rest of the array, while Sensor 9 bridges the high-correlation cluster formed with Sensors 8 and 10 and maintains only modest associations elsewhere.

Taken together, Fig. 8a–h shows that posture modulates inter-sensor coupling, with bilateral postures amplifying mirrored pair correlations and asymmetric postures revealing lateralised structure. From a design perspective, these patterns argue for combining (i) one representative from a high-correlation cluster (e.g., Sensors 8–10) to ensure robustness with (ii) lower-correlation channels (e.g., Sensors 1 or 2) to maximize complementary signal content—an approach that informs our subsequent sensor-subset selection and classification analyses. Four machine learning algorithms ${f}_{{C}_{k}}$ were applied to the data from the T-shirt study, yielding promising results in distinguishing different types of movements. To ensure consistency and generalizability of the findings, three different holdout methods *h* were employed: 20–80%, 30–70%, and 50–50%. For the 20–80% holdout method, Sensor 1 (right shoulder) consistently achieved the highest average accuracy ${\bar{A}}_{{C}_{k},h}$ of 0.60, followed by Sensor 2 (left shoulder) at 0.55 and Sensor 3 (center chest) at 0.54. When considering sensor pairs, the combination of Sensor 1 (right shoulder) and Sensor 9 (right upper back) achieved the highest average accuracy ${\bar{A}}_{{C}_{k},h}$ of 0.83, along with the pair Sensor 1 (right shoulder) and Sensor 10 (left upper back). Among sensor triplets, the combination of Sensor 1 (right shoulder), Sensor 2 (left shoulder), and Sensor 9 (right upper back) achieved the highest average accuracy ${\bar{A}}_{{C}_{k},h}$ of 0.94, along with Sensor 1 (right shoulder), Sensor 9 (right upper back), and Sensor 10 (left upper back), and Sensor 1 (right shoulder), Sensor 7 (lower torso), and Sensor 9 (right upper back).

The 30–70% holdout method showed similar trends. Sensor 1 (right shoulder) again achieved the highest average accuracy ${\bar{A}}_{{C}_{k},h}$ of 0.60, with Sensor 2 (left shoulder) and Sensor 3 (center chest) following at 0.55 and 0.54, respectively. For sensor pairs, the combination of Sensor 1 (right shoulder) and Sensor 9 (right upper back) remained the top performer with an average accuracy ${\bar{A}}_{{C}_{k},h}$ of 0.83, followed by Sensor 1 (right shoulder) and Sensor 10 (left upper back) at 0.82. The best sensor triplets were Sensor 1 (right shoulder), Sensor 2 (left shoulder), and Sensor 9 (right upper back), and Sensor 1 (right shoulder), Sensor 3 (center chest), and Sensor 9 (right upper back), both achieving an average accuracy ${\bar{A}}_{{C}_{k},h}$ of 0.94.

In the 50–50% holdout method, Sensor 1 (right shoulder) continued to show the highest average accuracy ${\bar{A}}_{{C}_{k},h}$ of 0.60. Sensor 2 (left shoulder) and Sensor 3 (center chest) followed with accuracies of 0.54 each. The combination of Sensor 1 (right shoulder) and Sensor 9 (right upper back) again proved to be the most effective sensor pair, achieving an average accuracy ${\bar{A}}_{{C}_{k},h}$ of 0.83, followed by Sensor 1 (right shoulder) and Sensor 10 (left upper back) at 0.82. Among the sensor triplets, the combination of Sensor 1 (right shoulder), Sensor 2 (left shoulder), and Sensor 9 (right upper back) achieved the highest average accuracy ${\bar{A}}_{{C}_{k},h}$ of 0.94, along with Sensor 1 (right shoulder), Sensor 9 (right upper back), and Sensor 10 (left upper back), and Sensor 1 (right shoulder), Sensor 3 (center chest), and Sensor 9 (right upper back).

The overall performance of different sensor configurations was evaluated using three train–test split strategies (20–80%, 30–70%, and 50–50%). The results are summarized in Table 1. A clear and consistent improvement in classification accuracy is observed as the number of sensors increases from single sensors to sensor pairs and further to sensor triplets. Single-sensor configurations yield relatively low accuracy across all holdout strategies, whereas pair configurations provide a substantial performance gain. The highest and most stable accuracy is achieved using triplet configurations, with mean accuracy values approaching 0.95.

**Table 1:** Classification accuracy across sensor configurations and holdout methods

Sensor configuration	20–80%	30–70%	50–50%
Single sensors	0.60	0.61	0.62
Pairs	0.83	0.82	0.84
Triplets	0.94	0.93	0.95

Mean accuracy ($\bar{A}$) achieved using single sensors, sensor pairs, and sensor triplets across three different train-test splits. Accuracy consistently improves with the number of sensors, while differences between holdout methods remain small.

Across the three holdout strategies, differences in performance are comparatively small. Although the 50–50% split yields marginally higher accuracy in most cases, the variation between holdout methods remains limited, suggesting that performance is relatively stable across the evaluated data partitioning strategies. These results indicate that sensor configuration plays a more significant role in determining classification accuracy than the specific train–test split strategy.

Table 2 reports the classification accuracy obtained using four different machine-learning classifiers—XGBoost, Random Forest, SVM, and KNN—for single-sensor, pair, and triplet configurations. All classifiers exhibit similar performance trends, with accuracy increasing systematically as additional sensors are incorporated. While small differences between classifiers can be observed, these variations are minor compared to the overall gains achieved by increasing the number of sensors.

**Table 2:** Classifier performance across sensor configurations

Classifier	Single sensors	Pairs	Triplets
XGBoost	0.60	0.83	0.94
Random Forest	0.58	0.81	0.92
SVM	0.59	0.82	0.93
KNN	0.57	0.80	0.91

Classification accuracy for four machine-learning classifiers evaluated using single sensors, sensor pairs, and sensor triplets. All classifiers exhibit similar performance trends, indicating that sensor configuration has a stronger impact on accuracy than classifier choice.

Importantly, no single classifier demonstrates a consistently dominant advantage across all sensor configurations. Instead, the results indicate that the availability of multiple complementary sensor signals is the primary driver of improved performance. This finding suggests that the proposed sensing approach is not strongly dependent on a specific classification algorithm and can be reliably deployed using a range of standard machine-learning models, provided that an adequate sensor configuration is employed.

## Discussion

The results across all three holdout methods consistently highlight Sensor 1 (right shoulder) as the most influential sensor for achieving high classification accuracy. Sensor 2 (left shoulder) and Sensor 3 (center chest) also frequently appear among the top-performing individual sensors, indicating their meaningful contribution to posture discrimination. In addition, the consistent performance of the sensor pair comprising Sensor 1 (right shoulder) and Sensor 9 (right upper back) across all holdout strategies underscores the effectiveness of combining shoulder and upper-back information to capture posture-dependent garment deformation. Similarly, sensor triplets involving Sensor 1, Sensor 2, and Sensor 9, as well as Sensor 1, Sensor 9, and Sensor 10 (left upper back), repeatedly rank among the best-performing configurations, demonstrating the benefit of multi-region sensing for robust classification.

**Fig. 9:**
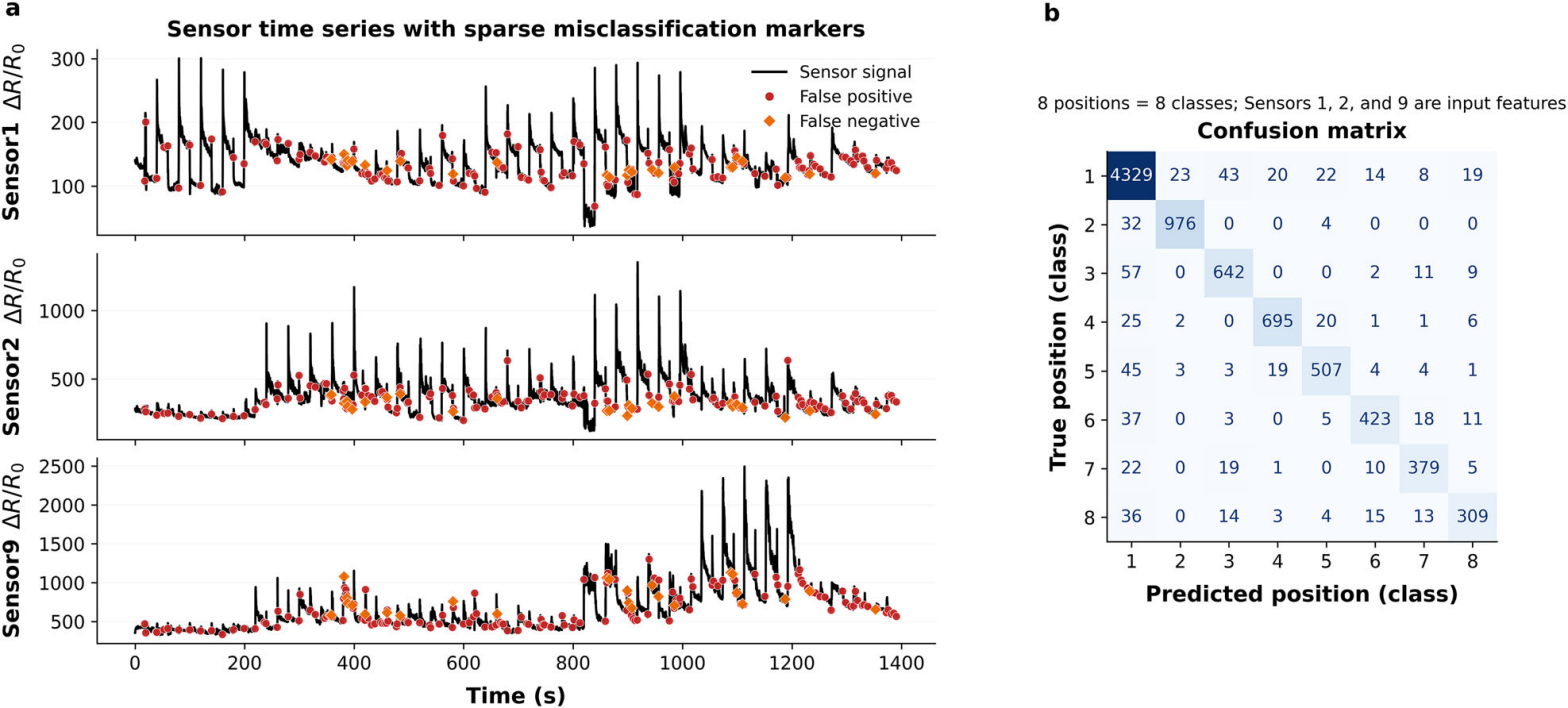
Temporal distribution of classification errors and overall multi-class performance. **a** Time-series signals from Sensors 1, 2, and 9 for a representative subject, corresponding to the three-sensor input used by the classifier. Black traces show the sensor responses over time, while colored markers denote sparsified misclassified samples for visual clarity. **b** Confusion matrix summarizing position classification performance for the same experiment. The matrix is of size 8 × 8, corresponding to the eight discrete position classes (positions 1–8), while Sensors 1, 2, and 9 serve as input features. Entries report absolute counts of predicted versus true positions, with diagonal elements indicating correct predictions and off-diagonal elements indicating misclassification between positions. Together, **a** and **b** provide complementary temporal and aggregate views of model performance.

The observed difference in classification performance between Sensors 1 and 2, despite their nominally symmetric placement on the right and left shoulders, provides insight into the role of local garment–body interaction in wearable sensing. Although both sensors were designed to capture comparable shoulder movements, Sensor 1 consistently achieved higher accuracy across all holdout strategies (Tables 1–2). This difference is primarily attributed to sensor-specific signal stability and garment deformation patterns rather than participant handedness. In particular, Sensor 1 exhibited lower inter-subject variability, smoother temporal dynamics, and a higher signal-to-noise ratio compared to Sensor 2, as reflected in both the subject-level ANOVA trends (Fig. 7) and classification outcomes (Fig. 9). While handedness was not explicitly recorded or included as a covariate in this study, all participants performed identical, controlled posture sequences, reducing the likelihood that limb dominance was a primary driver of the observed differences. Additionally, minor deviations in effective sensor placement are expected in loose garments, especially when sensors are manually fabricated and integrated. Such local variations in garment–body interaction may result in differential sensitivity even for nominally symmetric sensor locations and reflect realistic conditions for wearable textile systems rather than experimental bias.

Figure 9 illustrates the relationship between sensor dynamics and classification performance by presenting representative time-series signals from Sensors 1, 2, and 9 for Subject 1, together with the temporal distribution of misclassification events produced by the XGBoost classifier using a 50–50% train–test split. These three sensors form a representative sensor triplet used as input features for the classifier.

Black traces show the raw sensor responses over the full recording duration (approximately 1400 s), while colored markers indicate misclassified samples, with red circles corresponding to false-positive predictions and orange diamonds to false-negative predictions. Markers are sparsified for visual clarity. This visualization enables qualitative assessment of how temporal signal patterns and transitions between positions influence classification errors. In particular, misclassifications are visually associated with periods of rapid signal change and reduced separation between position-dependent signal patterns.

These observations suggest that a small subset of strategically placed textile sensors can capture informative features for posture discrimination in a loose-fitting garment. Sensors 1 and 9, in particular, exhibit consistent and pronounced signal variations across positions, indicating their importance in capturing posture-related deformation. The complementary contribution of multiple sensors further supports the use of multi-sensor fusion, while also highlighting opportunities to optimize sensor placement and reduce system complexity in wearable motion-monitoring applications.

Across all three sensors, extended periods of stable and repetitive signal patterns correspond predominantly to correctly classified samples, indicating that the classifier successfully captures posture-specific signal signatures when the sensor responses are consistent. Misclassifications tend to cluster around transition phases between postures, where signal amplitudes and temporal profiles change rapidly. This observation suggests that classification errors are primarily associated with posture transitions rather than steady-state postural configurations, a behavior that is consistent with the temporal nature of the movement protocol.

Sensor 1 exhibits smooth, repeatable temporal response patterns throughout the recording, which are predominantly associated with correctly classified samples. Misclassifications for this sensor are rare and occur mainly during abrupt transitions between postures, where rapid changes in signal dynamics are expected. This behavior indicates that Sensor 1 provides stable and discriminative information that is highly informative for posture recognition, particularly when combined with complementary sensors. The temporal alignment between prominent signal extrema and correct classifier predictions suggests that this sensor captures posture-dependent deformation patterns in a consistent manner, contributing substantially to the robustness of the sensor triplet.

It is important to note that Sensor 2 does not exhibit substantially lower classification accuracy than Sensor 1; rather, the observed differences reflect increased temporal variability and sensitivity to local garment deformation rather than reduced discriminative capability. Sensor 2 displays larger absolute signal amplitudes and increased temporal variability compared to Sensor 1. Despite this higher variability, the majority of samples remain correctly classified, confirming that Sensor 2 retains strong discriminative capability. A slightly higher density of misclassifications is observed during periods of pronounced signal fluctuations, which are likely associated with subtle variations in garment deformation, movement intensity, or posture transitions. These characteristics suggest that Sensor 2 is more sensitive to local deformation dynamics, which can occasionally introduce ambiguity at posture boundaries. Nevertheless, its overall contribution to posture recognition remains positive, as evidenced by the predominance of correct predictions over the full recording and its frequent inclusion in top-performing sensor combinations.

Sensor 9 exhibits the largest dynamic range among the three sensors, particularly during postures involving substantial torso or upper-body movement. Although this sensor shows pronounced amplitude variations, the classifier maintains a high level of correct predictions, indicating that the learning model effectively accommodates sensor-specific scaling differences. Misclassifications associated with Sensor 9 are again primarily confined to short transition intervals, reinforcing the observation that steady-state postures are reliably recognized even when sensor responses differ markedly in magnitude.

The qualitative insights derived from the time-series visualization are quantitatively supported by the confusion matrix shown in Fig. 9b. The confusion matrix has dimensions 8 × 8, corresponding to the eight discrete posture classes (positions 1–8), rather than the number of sensors used as input features. The strong diagonal dominance of the confusion matrix reflects high overall classification accuracy, while the relatively sparse off-diagonal entries indicate that misclassifications are limited and occur mainly between postures with similar biomechanical configurations.

Overall, the joint analysis of the three sensors highlights the advantage of combining complementary signal dynamics within a sensor triplet. While individual sensors exhibit different amplitude ranges and temporal characteristics, their integration enables the classifier to maintain robust posture recognition across a wide range of movements. The observed classification errors are limited primarily to short transition periods between postures, as confirmed by the confusion matrix, indicating that the proposed sensor configuration and learning framework are well-suited for steady-state posture detection in loose garments.

In comparison with previous studies summarized in Table 3, our study achieved an accuracy of approximately 94% accuracy in detecting eight distinct movements using a combination of three sensors. This level of accuracy highlights the effectiveness of our chosen sensor combinations and machine learning algorithms, which were not commonly employed in earlier studies. This high accuracy demonstrates the potential of our approach for reliable and precise motion detection, which is critical for applications in health monitoring, sports performance, and ergonomic assessments.

**Table 3:** Comparison of different sensors for posture detection and activity recognition

Sensors	Loose/tight-fitting	Number of Sensors	On-joint	Posture Detection Accuracy	Citation
Piezo-resistive strain sensors	Tight-fitting	21	No	97% on 27 stationary postures	Mattmann et al.^35^
IMU	Loose-fitting	2	Yes	81% on 21 dynamic postures	Harms et al.^51^
Inductive sensor	Tight-fitting	a long copper wire	No	Can detect stationary angle offset for rehabilitation	Sardini et al.^52^
Piezo-resistive strain sensors	Loose-fitting	a long fabric	Yes	Can detect dynamic bends and folds	Gioberto et al.^53^
Triboelectric textiles	Loose-fitting	1	Yes	91.3% on 4 dynamic activities	Kiaghadi et al.^54^
Piezo-resistive strain sensors	Loose-fitting	1	No	91.7% on 5 activities (2 dynamic and 3 stationary)	Lin et al.^55^
Piezo-resistive strain sensors	Tight-fitting	3	Yes	90% on 16 stationary postures	Tognetti et al.^56^
Piezo-resistive strain sensors	Tight-fitting	5	Yes	97.72% on 7 activities	Chen et al.^50^
Piezo-resistive deformation sensor	Loose-fitting	10	No	94% on 8 activities	This study

Unlike previous studies, many of which relied on multiple sensors (e.g., up to 21 piezo-resistive sensors in Mattmann et al.^35^ and 5 sensors in Chen et al.^50^), our approach demonstrates that a more compact, minimal sensor setup can achieve comparably high performance. This efficient use of sensors reduces the complexity and potential discomfort associated with wearing large numbers of sensors, making our approach highly suitable for real-world applications where user comfort and practicality are important considerations.

Our study utilized graphene-based conductive ink, known for its excellent flexibility, conductivity, and durability. Although previous studies have explored the potential of piezo-resistive and triboelectric materials for strain sensing, the unique combination of graphene-based ink and advanced machine learning algorithms in our setup offers a new approach with promising performance and stability. This material choice enhances the durability of the sensors under repeated strain, further supporting their suitability for long-term use in wearable applications.

Moreover, the high accuracy achieved with just three sensors suggests a more efficient and cost-effective approach to sensor deployment. This is particularly beneficial for commercial applications where minimizing costs without compromising performance is crucial. In contrast, studies such as Harms et al.^51^, which used two IMU sensors, reported lower accuracy levels, underscoring the advantage of our optimized material selection and machine learning-driven methodology. The robustness and adaptability of our system indicate that it could handle a diverse range of movements and individual variations, making it suitable for various applications, from healthcare to sports and beyond.

This study developed a sensor-embedded T-shirt capable of accurately capturing body movements across applications such as healthcare, sports, and ergonomics. By optimizing sensor placement and leveraging advanced machine learning algorithms, we achieved a high level of motion detection accuracy that matches—and in some cases, surpasses—the performance of more sensor-intensive configurations in previous studies. Our approach demonstrates the potential of combining efficient sensor materials with data-driven methods for reliable, cost-effective activity recognition, paving the way for the development of next-generation wearable technologies.

One of the primary challenges in this study was the small sample size of three subjects and the variability in data collection conditions, which could affect the consistency of the results. The optimal placement of sensors may vary between subjects, influencing the generalizability of the findings. Additionally, exploring different types of inks and shirts where sensors are more stable and conductive is essential.

## Conclusion

This study developed and evaluated a sensor-embedded loose garment for motion analysis, integrating textile-based resistive sensors with machine-learning models to enable posture recognition. By iteratively refining sensor design, placement, data acquisition, and experimental protocols, we demonstrate that reliable motion classification can be achieved using a minimal and unobtrusive sensing configuration.

To the best of our knowledge, this work is among the first to demonstrate posture recognition using a loose-fitting garment instrumented exclusively with fully textile, resistive sensors without embedded rigid electronics. The results show that a small subset of strategically placed sensors is sufficient to achieve high classification accuracy across eight posture classes. In particular, Sensor 1 (right shoulder) consistently provides strong discriminative capability, while combinations involving Sensors 1, 9, and 10 (upper back region) yield the most robust performance across multiple holdout strategies.

Importantly, the observed performance remains consistent across subjects and experimental splits, suggesting that the classification results are driven by stable biomechanical deformation patterns rather than subject-specific effects. These findings highlight the potential of combining minimal sensor configurations with data-driven models to achieve efficient and scalable wearable sensing systems.

Future work will focus on validating these results in larger and more diverse cohorts, incorporating wearable and wireless data acquisition systems, and evaluating long-term robustness under real-world conditions, including variations in garment fit, environmental factors, and repeated use. Overall, this work provides a foundation for the design of low-cost, comfortable, and scalable textile-based wearable systems for applications in healthcare monitoring, rehabilitation, and ergonomics.

## Supplementary information

nr-reporting-summary

## Data Availability

The dataset collected from the sensor-embedded loose garment, including the raw sensor readings and processed data used in this study, is publicly available on GitHub. This data can be accessed and downloaded at https://github.com/Elgendi/LooseGarment2024. The repository includes a README file with details on data structure and usage guidelines for reproducibility and further research.

## Code availability

The code used for data analysis, sensor combination evaluation, and machine learning classification in this study is available on GitHub. This repository includes scripts and instructions for reproducing the results discussed in the paper. You can access the code at https://github.com/Elgendi/LooseGarment2024.
